# General anaesthesia for insertion of an automated implantable cardioverter defibrillator in a child with Brugada and autism

**DOI:** 10.4103/0019-5049.72648

**Published:** 2010

**Authors:** Shwetal Goraksha, Sneha Bidaye, Supriya Gajendragadkar, Jitendra Bapat, Manju Butani

**Affiliations:** Department of Anaesthesiology, P.D. Hinduja Hospital and Research Centre, Mumbai, India

**Keywords:** AICD, autism, Brugada syndrome

## Abstract

A 14-year-old autistic boy presented with acute gastroenteritis and hypotension. The electrocardiogram showed a ventricular fibrillation rhythm – he went into cardiorespiratory arrest and was immediately resuscitated. On investigation, the electrocardiogram showed a partial right bundle branch block with a “coved” pattern of ST elevation in leads v_1_–v_3_. A provisional diagnosis of Brugada syndrome was made, for which an automated implantable cardioverter defibrillator (AICD) implantation was advised. Although the automated implantable cardioverter defibrillator implantation is usually performed under sedation, because this was an autistic child, he needed general anaesthesia. We performed the procedure uneventfully under general anaesthesia and he was discharged after a short hospital stay.

## INTRODUCTION

Brugada syndrome was first described as a separate clinical entity in 1992 by Brugada and Brugada.[1] Patients suffering from Brugada syndrome have a characteristic electrocardiogram pattern of right bundle branch block (RBBB) with ST elevation in leads V_1_–V_3_ and are at a high risk for malignant dysrhythmia and cardiac arrest. Many factors during general anaesthesia (medications, bradycardia and temperature changes) could precipitate malignant dysrhythmia. We present a case of a 14-year-old male with Brugada syndrome and autism that was posted for an automated implantable cardioverter defibrillator under general anaesthesia.

## CASE REPORT

A 14-year-old child, weighing 42 kg, was admitted with acute gastroenteritis and hypotension. The electrocardiogram showed ventricular fibrillation and he went into cardiorespiratory arrest; he was revived with immediate cardiopulmonary cerebral resuscitation and shifted to the intensive care unit with inotropic and ventilator support.

The 12-lead electrocardiogram showed a RBBB with a “coved” pattern of ST segment elevation in leads V_1_–V_3_. Cardiac enzymes were not elevated and 2D echocardiography showed normal ventricular function, with no underlying structural cardiac problem. A provisional diagnosis of Brugada syndrome was made.

Once the patient was stable, we allowed a “Flecainide challenge test” under sedation with an intravenous bolus of 2 mg/kg of flecainide. This resulted in 50% accentuation of ST segment elevation in leads v3 and v4, which is consistent with the features of Brugada syndrome. He was posted for an automated implantable cardioverter defibrillator insertion.

A detailed history revealed that the patient was autistic and had a documented non-progressive congenital myopathy. After clinically assessing the child pre-operatively, we obtained the necessary investigations. Because he was autistic and uncooperative, we decided to perform the procedure under general anaesthesia.

A written, informed consent was taken from the parents. During the procedure, the saturation (SpO_2_), end tidal CO_2_, non-invasive blood pressure, temperature and electrocardiogram readings were monitored and an external defibrillator connected to disposable defibrillation pads was placed on the patient. After establishing an intravenous access, he was given midazolam 1 mg. Anaesthesia was induced with 1.5 mg/kg propofol and 1 mcg/kg fentanyl. Atracurium 0.5 mg/kg was used to intubate the patient. Anaesthesia was maintained with a Datex Ohmeda anaesthesia machine from which we had already removed the vaporizers and flushed the machine with oxygen. We used an oxygen:air mixture with propofol infusion and atracurium. The procedure was uneventful and there was no bradycardia or ventricular fibrillation unrelated to electrophysiological stimulation. At the end of the procedure, neostigmine 0.05 mg/kg and glycopyrrolate 0.008 mg/kg were used to reverse the neuromuscular blockade and the patient was extubated. He was monitored in the intensive care unit for 48 h and discharged following an uneventful hospital stay.

## DISCUSSION

Brugada syndrome is genetic, characterized by abnormal electrocardiogram findings, and is also known as Sudden Unexpected Death Syndrome or Sudden Unexpected Nocturnal Death Syndrome.

The average age of presentation is 40 years, but can vary from 2 to 77 years.[2] It is more common in men, with a higher prevalence in the Asian populations. An estimated 4% of all sudden deaths and at least 20% of sudden deaths in patients with structurally normal hearts are due to the syndrome.[3] This condition is inherited in an autosomal-dominant pattern, with incomplete penetrance. It should be suspected in any cardiac arrest or syncope of unknown origin with or without ventricular fibrillation. However, some patients remain asymptomatic and diagnosis is suggested by a routine electrocardiogram showing ST-segment elevation in leads V_1_–V_3_. In about 20% of the patients, atrial fibrillation is an associated arrhythmia.[4]

It is an example of a channelopathy, a disease caused by an alteration in the transmembrane ion currents that together constitute the cardiac action potential. Specifically, in 10–30% of the cases, mutations occur in the SCN5A gene that encodes the cardiac voltage-gated sodium channel.[5,6] Loss of function mutations in this gene lead to a loss of the action potential dome of some epicardial areas of the right ventricle. This results in transmural and epicardial dispersion of repolarization. The transmural dispersion underlies ST-segment elevation and the development of a vulnerable window across the ventricular wall, whereas the epicardial dispersion of repolarization facilitates the development of phase 2 re-entry, which generates a phase 2 re-entrant extrasystole that precipitates ventricular tachycardia and/or fibrillation, which often results in sudden cardiac death.

**Figure 1:**
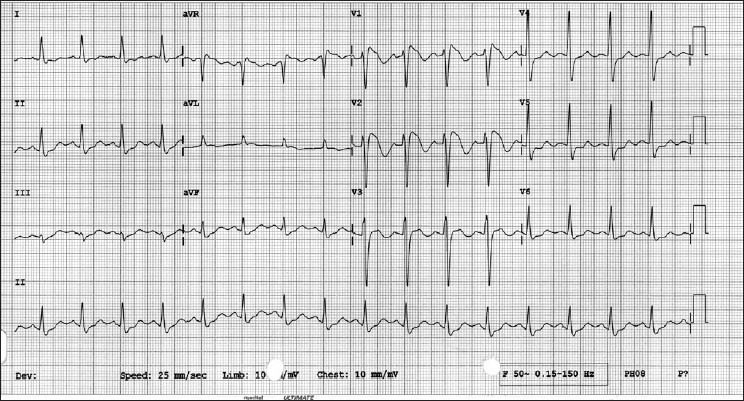
Typical ECG findings in Brugada syndrome

Typical electrocardiogram findings include a RBBB with ST elevation in the precordial leads characteristically “coved or saddleback,” with or without the terminal S waves in the lateral leads and associated with a typical RBBB, with no prolongation of QT interval. Prolongation of PR interval is also frequently seen [Figure 1].

Brugada syndrome has three different electrocardiogram patterns. Type 1 has a coved-type ST elevation with at least 2-mm J-point elevation, a gradually descending ST segment and a negative T-wave. The electrocardiogram picture in this study shows the typical Type 1 pattern.

Type 2 has a saddle back pattern with a least 2-mm J-point elevation and at least 1-mm ST elevation, with a positive or biphasic T-wave. The Type 2 pattern can occasionally be seen in healthy subjects.

Type 3 has a saddle back pattern with <2-mm J-point elevation and <1-mm ST elevation, with a positive T-wave. Type 3 pattern is not uncommon in healthy subjects.

Increased serum potassium and calcium levels may generate a similar electrocardiogram pattern. Laboratory markers such as creatine kinase-MB (CK-MB) and troponin rule out an acute coronary syndrome. Echocardiography and/or magnetic resonance imaging should be performed to exclude structural abnormalities.

Drug challenge with sodium channel blockers is a standard provocative test used to unmask Brugada syndrome.[7] We used flecainide infused at a dose of 2 mg/kg over 10 min, with continuous cardiac monitoring. It produced an accentuation of ST segment elevation in the precordial leads.[7,8] The sensitivity and specificity of these tests have not yet been confirmed.

The electrocardiogram readings fluctuate depending on the autonomic balance.[9] Adrenergic stimulation decreases the ST elevation and drugs such as isoproterenol ameliorate the electrocardiogram manifestations. Fever, vagal stimulation, administration of class Ia, Ic and III drugs and neostigmine also accentuate the ST segment elevation. Acetylcholine, beta antagonists and nicorandil may interfere with the ionic conditions and exacerbate the manifestations.

Local anaesthetics, especially bupivacaine, given by any route (e.g., epidural), which cause a sudden rise in the serum concentration can unmask Brugada syndrome.[10] Lignocaine, a class IIb anti-arrhythmic drug has no such effect and can be safely used.[9]

Our patient had documented congenital non-progressive myopathy and, therefore, we avoided inhalation agents as a precaution against malignant hyperthermia. Isoflurane should also be avoided in patients with prolonged QT interval.[11]

Our patient had received atracurium earlier without any complications and was therefore our drug of choice for neuromuscular blockade. Administration of neostigmine is also known to elevate the ST segment. However, in our patient, it did not cause any detectable cardiac arrhythmia.[12]

The only treatment is the insertion of an automated implantable cardioverter defibrillator, which continuously monitors the heart rhythm and defibrillates the patient if ventricular fibrillation is noted. No pharmacological therapy is beneficial.

Some recently performed studies had evaluated the role of quinidine, a Class Ia anti-arrhythmic drug, for decreasing VF episodes occurring in this syndrome. Quinidine was found to decrease the number of VF episodes and correcting spontaneous electrocardiogram changes, possibly via inhibiting the Ito channels.

Brugada is an increasingly recognized syndrome. The importance of detecting Brugada is due to its high prevalence in the Asiatic young population. Brugada syndrome may be a significant cause of death, aside from accidents, in men under 40 years. The true incidence is not known due to the reporting biases. Although there is a strong population dependence, an estimated 4% of all sudden deaths and at least 20% of the sudden deaths in patients with structurally normal hearts are due to the syndrome. Those with the syndrome have a mean age of sudden death of 41±15 years.[3]

We, as anaesthesiologists, have to take care when using alpha agonists and neostigmine in such patients and avoid class I anti-arrhythmic drugs altogether. These patients should be monitored in a high-dependency unit post-operatively so that any cardiac arrhythmias can be timely detected and treated.
